# [^18^F]-AV-1451 binding profile in chronic traumatic encephalopathy: a postmortem case series

**DOI:** 10.1186/s40478-019-0808-1

**Published:** 2019-10-28

**Authors:** Marta Marquié, Cinthya Agüero, Ana C. Amaral, Alberto Villarejo-Galende, Prianca Ramanan, Michael Siao Tick Chong, Nil Sáez-Calveras, Rachel E. Bennett, Eline E. Verwer, Sally Ji Who Kim, Maeva Dhaynaut, Victor E. Alvarez, Keith A. Johnson, Ann C. McKee, Matthew P. Frosch, Teresa Gómez-Isla

**Affiliations:** 1 MassGeneral Institute for Neurodegenerative Disease, Charlestown, MA USA; 2 Department of Neurology, Massachusetts General Hospital, WACC Suite 715 15th Parkman St, Boston, MA 02114 USA; 3 Department of Neurology, Hospital Universitario Doce de Octubre, CIBERNED, Madrid, Spain; 4 Gordon Center for Medical Imaging, Division of Nuclear Medicine and Molecular Imaging, Department of Radiology, Massachusetts General Hospital, Boston, MA USA; 5 AP-HP, Department of Nuclear Medicine, Pitié-Salpêtrière Hospital, Sorbonne University, UPMC Paris 06, CNRS UMR 7371, INSERM U1146, 75013 Paris, France; 6 Departments of Neurology and Pathology, Boston University School of Medicine, Boston, MA USA; 7 Department of Pathology and Laboratory Medicine, VA Boston Healthcare System, Boston, MA USA; 8 Boston University Alzheimer’s and CTE Center, Boston, MA USA; 9 C.S. Kubik Laboratory for Neuropathology, Massachusetts General Hospital, Boston, MA USA

**Keywords:** Chronic traumatic encephalopathy, [^18^F]-AV-1451, Flortaucipir, Tau, PET, Autoradiography

## Abstract

**Introduction:**

Chronic traumatic encephalopathy (CTE) is a tauopathy associated to repetitive head trauma. There are no validated in vivo biomarkers of CTE and a definite diagnosis can only be made at autopsy. Recent studies have shown that positron emission tomography (PET) tracer AV-1451 (Flortaucipir) exhibits high binding affinity for paired helical filament (PHF)-tau aggregates in Alzheimer (AD) brains but relatively low affinity for tau lesions in other tauopathies like temporal lobal degeneration (FTLD)-tau, progressive supranuclear palsy (PSP) or corticobasal degeneration (CBD). Little is known, however, about the binding profile of this ligand to the tau-containing lesions of CTE.

**Objective:**

To study the binding properties of [^18^F]-AV-1451 on pathologically confirmed CTE postmortem brain tissue samples.

**Methods:**

We performed [^18^F]-AV-1451 phosphor screen and high resolution autoradiography, quantitative tau measurements by immunohistochemistry and Western blot and tau seeding activity assays in brain blocks containing hippocampus, superior temporal cortex, superior frontal cortex, inferior parietal cortex and occipital cortex from 5 cases of CTE, across the stages of disease: stage II-III (*n* = 1), stage III (*n* = 3), and stage IV (*n* = 1). Importantly, low or no concomitant classic AD pathology was present in these brains.

**Results:**

Despite the presence of abundant tau aggregates in multiple regions in all CTE brains, only faint or no [^18^F]-AV-1451 binding signal could be detected by autoradiography. The only exception was the presence of a strong signal confined to the region of the choroid plexus and the meninges in two of the five cases. Tau immunostaining and Thioflavin-S staining ruled out the presence of tau aggregates in those regions. High resolution nuclear emulsion autoradiography revealed the presence of leptomeningeal melanocytes as the histologic source of this *off-target* binding. Levels of abnormally hyperphosphorylated tau species, as detected by Western Blotting, and tau seeding activity were both found to be lower in extracts from cases CTE when compared to AD.

**Conclusion:**

AV-1451 may have limited utility for in vivo selective and reliable detection of tau aggregates in CTE. The existence of disease-specific tau conformations may likely explain the differential binding affinity of this tracer for tau lesions in different tauopathies.

## Introduction

Chronic traumatic encephalopathy (CTE) is a neurodegenerative disorder associated with repetitive traumatic head injuries and characterized by the deposition of hyperphosphorylated tau aggregates in the brain [5, 6]. This condition, originally observed in boxers “punch drunk” [23], “dementia pugilistica” [28], has since been described in players of contact sports [13] and military personnel exposed to blast injuries [30]. The clinical picture of CTE includes progressive behavioral and cognitive changes, including irritability, aggression, depression and memory loss, with onset years or decades after brain injury [25], that can eventually progress to dementia [11, 12].

The current clinical criteria of CTE lack specificity and the definitive diagnosis of this condition can only be established by neuropathologic examination*.* A set of consensus neuropathological criteria for CTE were defined in 2016, which emphasize that tau-containing lesions in CTE differ from those of other tauopathies such as Alzheimer disease (AD), progressive supranuclear palsy (PSP) or corticobasal degeneration (CBD) [24]. The pathognomonic lesions for CTE consist of tau aggregates in neurons, astrocytes and cell processes around small vessels in an irregular pattern in the depths of the cortical sulci [24]. The presence of other neurodegenerative lesions such as TAR DNA binding protein 43 (TDP-43) inclusions and β-amyloid pathology (including plaques and amyloid angiopathy) is also a frequent concomitant finding in CTE [24, 26]. Four progressive stages of CTE have been described according to the abundance and distribution of tau lesions [26]. Tau aggregates in CTE contain all six isoforms with presence of both 3 (3R) and 4 (4R) repeats of the microtubule binding domain, similar to AD but distinct from most other tauopathies [34]. Despite this similarity, it has recently been demonstrated by electron cryomicroscopy (cryo-EM) that tau filament conformation in CTE differs from that of tau filaments present in classic neurofibrillary tangles (NFTs) of AD [9, 10].

There is great interest in developing novel biomarkers for CTE to estimate the prevalence of this disorder in at-risk populations, improve diagnostic accuracy, allow disease progression tracking, and assess treatment response. Several positron emission tomography (PET) tracers designed for detection of tau aggregates in the human living brain have been developed in the past few years. After a number of early failures, [^18^F]-AV-1451 (alternatively called flortaucipir and previously [^18^F]-T807) was reported [41] as the first promising ligand for imaging tau in AD. Increased in vivo [^18^F]-AV-1451 uptake has been observed in AD patients compared to cognitively normal controls (CTL) in cortical regions known to contain NFTs [1, 3, 4, 14, 17, 32, 35, 39]. The usefulness of [^18^F]-AV-1451 as a biomarker in other tauopathies such as frontotemporal lobar degeneration (FTLD)-tau including Pick’s disease (PiD), PSP, and CBD, however, is more controversial. Some authors reported increased in vivo [^18^F]-AV-1451 retention in patients clinically diagnosed with non-Alzheimer (non-AD) tauopathies in regions that are expected to contain tau lesions while others noticed in vivo binding patterns nearly indistinguishable from those in normal controls [1, 3, 4]. Several groups, including our own have demonstrated, using autoradiography approaches in postmortem brain tissue samples, that [^18^F]-AV-1451 has a significantly higher affinity for tau aggregates in the form of NFTs in AD compared to tau aggregates in non-AD tauopathies [19–21, 33]. Importantly, [^18^F]-AV-1451 also exhibits strong *off-target* binding to neuromelanin (in pigmented brainstem regions) and melanin (in leptomeninges). The former of these affinities explains the nearly universal elevated in vivo retention observed in the substantia nigra of elderly individuals regardless of their pathological diagnosis [21]. There is additional *off-target* binding in areas of intraparenchymal hemorrhage, although to a lesser degree [21]. The underlying pathology of this tracer’s in vivo uptake frequently detected in other brain regions that do not typically contain tau aggregates in AD, such as basal ganglia, is still not yet well understood.

Only a few studies using [^18^F]-AV-1451 PET in clinically diagnosed CTE subjects have been published to date [8, 29, 36]. Results from those early reports have suggested that this tau tracer may serve as an in vivo surrogate marker for tau-containing aggregates in this condition. To date, no [^18^F]-AV-1451 imaging-postmortem correlation studies in pathologically confirmed CTE cases have been published. The aim of our study was to investigate [^18^F]-AV-1451 binding patterns in pathologically confirmed CTE tissue using phosphor screen and high-resolution autoradiography and correlate those findings with quantitative tau measurements as reported by immunohistochemistry, Western blotting, and tau seeding activity in the same samples. Our results show that [^18^F]-AV-1451 exhibits relatively low binding affinity for tau aggregates in CTE suggesting that this tracer may have limited utility for the in vivo selective and reliable detection of tau aggregates which serve as the neuropathologic hallmark of this condition.

## Material and methods

### Brain tissue samples

Five cases from the Boston University (BU) Alzheimer’s Disease Research Center (ADRC) Brain Bank with a neuropathological diagnosis of CTE were selected for this study. Tissue collection and use were approved by the local Institutional Board. The neuropathological processing followed the procedures previously established by the BU ADRC Brain Bank [38]. Paraffin-embedded sections were stained with Luxol fast blue, hematoxylin and eosin, Bielschowsky silver, AT8, amyloid-β (Aβ), α-synuclein, ubiquitin, TDP-43, SMI-31 and SMI-34 using methods described previously [25]. Diagnostic evaluation was performed in accordance with published guidelines for neurodegenerative diseases [2, 16, 27]. Neuropathological diagnoses were made by a BU ADRC Brain Bank neuropathologist without any knowledge of the subject’s clinical histories and was confirmed by two other neuropathologists.

Frozen brain tissue blocks containing hippocampus (HPC), superior temporal cortex (TC), superior frontal cortex (FC), inferior parietal cortex (PC) and occipital cortex including calcarine cortex (OC) were analyzed. Demographical and neuropathological information for each case is shown in Table 1. Cases were classified according to the recently described CTE staging [26] into stages I-IV. The tissue blocks were sectioned in a freezing cryostat (Thermo-Shandon SME Cryostat) into 10-μm-thick slices and used for immunohistochemistry (IHC) and phosphor screen and nuclear emulsion autoradiography. Fresh frozen homogenates prepared from the same tissue blocks were used to quantify tau contents by Western Blot and to measure tau seeding activity by sensitive in vitro seeding assays. Fresh frozen homogenates containing the same regions of interest (ROIs) from 15 additional cases (including 9 pathologically-confirmed AD cases and 6 control cases free of pathology) from the Massachusetts ADRC where also included in the Western Blot and tau seeding assays for comparison.

**Table 1:** **Table 1** Demographic and neuropathologic characteristics

Case N°	Pathological diagnosis	Age at death (years)	Gender	Braak & Braak (NFTs)	CERAD score (neuritic plaques)	NIA-Reagan Institute criteria
1	CTE (CTE stage III)	46	M	III	none	LP
2	CTE (CTE stage IV)	65	M	II	A	LP
3	CTE (CTE stage III)	56	M	II	none	LP
4	CTE (CTE stage II-III)	25	M	0	none	LP
5	CTE (CTE stage III)	58	M	III	none	LP
6	AD	87	F	V	Frequent	IP
7	AD	71	M	V	Moderate	IP
8	AD	96	F	V	Frequent	HP
9	AD	82	F	V	Moderate	HP
10	AD	79	M	V	Frequent	HP
11	AD	66	M	V	Moderate	HP
12	AD	69	F	VI	Frequent	HP
13	AD	66	F	VI	Frequent	HP
14	AD	66	F	VI	Frequent	HP
15	CTL	76	F	I	Sparse	LP
16	CTL	97	F	I	Sparse	LP
17	CTL	81	M	I	none	LP
18	CTL	59	F	I-II	Moderate	LP
19	CTL	91	M	II	none	LP
20	CTL	101	F	II	Moderate	LP

*Abbreviations*: *CTE* Chronic traumatic encephalopahty, *AD* Alzheimer’s disease, *CERAD* Consortium to Establish a Registry for Alzheimer´s Disease, *CTL* Control, *F* Female, *HP* High probability of AD, *IP* Intermediate probability of AD, *LP* Low probability of AD, *M* Male, *NFT* Neurofibrillary tangles, *NIA* National Institute of Ageing

### [^18^F]-AV-1451 phosphor screen and high resolution autoradiography

10-μm-thick frozen tissue sections containing the ROIs were used to perform [^18^F]-AV-1451 phosphor screen and high resolution autoradiography experiments following our previously published protocols [21]. In brief, sections were fixed in 100% methanol at room temperature (RT) for 20 min and transferred to a bath containing high specific activity [^18^F]-AV-1451 in 10 mM PBS with a radioactivity concentration of approximately 20 μCi/ml. Adjacent sections were placed in an identical bath except that unlabeled AV-1451 was added to yield 1 μM chemical concentration, a blocking condition sufficient to saturate essentially all specific binding sites of tau [21]. After incubation for 60 min, sections were removed from radioactivity solutions and washed to remove unbound radiotracer. Wash solutions and incubation times were: 10 mM PBS for 1 min, 70% ethanol/30% PBS for 2 min, 30% ethanol/70% PBS for 1 min, and lastly 100% 10 mM PBS for 1 min. Sections were then air dried before transferring to a storage phosphor screen (MultiSensitive Phosphor Screen, PerkinElmer Life and Analytic Sciences, Shelton, CT) that had been photobleached immediately prior by exposure on a white light box for a minimum of 15 min. Sections and phosphor screen were enclosed in aluminum film cassette and set in a dark area. Under dim lighting conditions, the cassette was opened and the slides removed from the exposed screen, which was mounted to the carousel of imaging system (Cyclone Plus Storage Phophor Scanner, PerkinElmer Life and Analytic Sciences). Scanning of screens was controlled by the manufacturer’s OptiQuant software package using the highest available resolution of 600 dpi (approximately 42 μm sampling interval). Digital images were saved in uncompressed form at full resolution and pixel depth. Images from adjacent brain slices incubated in the unblocked (high specific activity [^18^F]-AV-1451 only) and blocking [^18^F]-AV-1451 plus 1 μM unlabeled AV-1451) conditions were compared to determine total and non-specific binding of [^18^F]-AV-1451 in the tissue. All experiments were run in triplicate on adjacent tissue sections.

To rule out the possibility that the ethanol washing steps used in the above autoradiography protocol may have removed some weaker tracer binding, parallel experiments in comparable tissue samples incubated with [^18^F]-AV-1451 with a radioactivity concentration of approximately 1 μCi/ml and avoiding the use of ethanol in the washing conditions were also conducted. All assays were done in triplicate on adjacent tissue sections.

To obtain autoradiographic information at cellular resolution level, frozen cryostat sections, adjacent to those used for phosphor screen autoradiography, were coated with liquid photographic emulsion, then immunostained using appropriate primary antibodies - anti-tau PHF1 (1:100, mouse, kind gift of Dr. Peter Davies), anti Aβ (1:50, rabbit, IgG Affinify Purify, IBL) or anti TDP-43 (1:100, rabbit, Protein Tech) – and counterstained with H&E following our previously published protocol [20].

### Tau burden quantification by immunohistochemistry

10-μm-thick frozen tissue sections containing the five ROIs and adjacent to those used in autoradiography experiments were stained with PHF-1 antibody (1:100, mouse, kind gift of Dr. Peter Davies) and analyzed with an upright Olympus BX51 microscope (Olympus, Denmark) using the CAST software (Visiopharm, 2004, Denmark). Each ROI was drawn in the corresponding slide at 1.25x magnification, and then a systematic random sampling was applied using the software’s optical disector probe at 10x magnification (meander sampling 20%). A threshold of optical density was obtained in each microphotograph using ImageJ (National Institute of Health). Manual editing in each field eliminated artifacts. Tau pathology burden, defined as total percentage (%) of area covered by PHF-1 immunostaining, was calculated in each ROI.

### Measurements of soluble tau in synaptoneurosomal fractions by Western blot

Synaptoneurosomal fractions from the five ROIs were obtained from the same tissue blocks used in immunohistochemistry and autoradiography experiments to measure soluble tau content by Western blot, following our previously published protocol [20]. Human tau (Dako, A0024) and PHF-1 (kind gift of Dr. Peter Davies) antibodies were used to assess levels of total tau and hyperphosphorilated-tau, respectively. Briefly, tissue samples were homogenized in Buffer A (25 mM HEPES 7.5, 120 mM NaCl, 5 mM KCL, 1 mM MgCl2, 2 mM CaCl2, 1 mM DTT) supplemented with Phosphatase Inhibitor Cocktail tablets (Roche, 04906845001) and Protease Inhibitor Cocktail tablets (Roche, 11,697,498,001). The homogenate was filtered through 2 Millipore Nylon 80 μm filters after the addition of 0.6 mL Buffer A and 200 μL of homogenate was separated. Two hundred microliter of distilled H_2_O and 70 μL of 10% SDS were added to the homogenate and passed through a 27½ G needle 3 times. The remaining homogenate was filtered again through PALL Acrodisc Syringe 5 μm Filters after the addition of 1 mL Buffer A, and centrifuged at 1000 g for 10 min at 4 °C. The supernatant was then separated and ultracentrifuged at 100,000 g for 45 min, and the pellet was resuspended in 200 μL Buffer B (50 mM Tris, 1.5% SDS, 1 mM DTT). The resuspended pellet and the separated homogenate were then boiled for 5 min, centrifuged for 15 min, and the supernatants were collected as synaptoneurosome (SNS) and total fractions, respectively.

SNS fractions from each ROI were electrophoresed in MES SDS Running Buffer (Novex, NP0002) using 4–12% Bis-Tris Novex gels (Invitrogen, #MAN0003679). Protein was then transferred onto nitrocellulose membranes and blocked for an hour at room temperature using Odyssey Blocking Buffer (LiCor, 927–40,000). Membranes were probed with human tau (Dako, A0024) and PHF-1 (kind gift of Dr. Peter Davies) antibodies to detect content of total tau and phospho-tau, respectively. GAPDH (Millipore, AB2302) was used as loading control for protein normalization. LiCor secondary antibodies (IR Dye 680RD Donkey anti-chicken 926–68,075, IR Dye 800 CW Donkey anti-rabbit 926–32,213, IR Dye 800CW Donkey anti-mouse 926–32,212) were then used to visualize bands with the Odyssey Infrared Imaging System (V3.0). ImageStudio was used to quantify the bands of interest by drawing equal size rectangles around individual bands of interest. Background subtraction was applied by taking the median of the area three pixels to the left and right of each band of interest and subtracting that value from the measured signal.

### Tau seeding assays

PBS fractions obtained from fresh frozen total homogenates from the five ROIs were used to perform tau seeding activity assays. Equivalent samples from AD (*n* = 9) and control cases free of tau pathology (*n* = 6) were also analyzed for comparison. In vitro tau seeding activity was measured as previously described elsewhere [15]. In short, HEK293 cells stably expressing TauRD^P301S^ fused with cyan fluorescent protein (CFP) and TauRD^P301S^-yellow fluorescent protein (YFP) (Tau RD P301S FRET Biosensor (ATCC CRL^R^-3275™)) were plated at 40,000 cells per well in poly-D-lysine coated black clear-bottom 96-well plates (Corning). The following day, cells were transduced with a total of 50 μl of 2 μg/well PBS-soluble frozen brain lysate (3,000 *g*) plus 7% lipofectamine 2000 transfection reagent (#11668–019, Invitrogen) in Opti-MEM (#11058–021, Life technologies), after the lysate and lipofectamine complex had incubated at room temperature for 20 min. The cells were left to incubate at 37 °C with the lysate-lipofectamine complex for 41 h. Cells were then removed from the plate using Trypsin-EDTA and transferred to a 96-well U-bottom plate (Corning) with chilled DMEM 10% FBS media. Cells were pelleted at 1,800 *g* using a plate spinner, fixed with 2% paraformaldehyde (PFA, #15714-S, Electron Microscopy Sciences) in the dark for 10 min at 4 °C, and pelleted again. Cells were resuspended in cold PBS and immediately run on the MACSQuant VYB flow cytometer (Miltenyi). CFP and FRET were measured on the flow cytometer by exciting the HEK293 cells with a 405 nm laser and then reading fluorescence at 405/50 nm and 525/50 nm filters, respectively. Using the MACSQuantify flow cytometry software (Miltenyi), the integrated FRET density (IFD) was calculated by multiplying the % number of FRET-positive tau aggregates by the mean fluorescence intensity of FRET-positive tau aggregates. This value was then normalized by the value for the cells that were treated only with the lipofectamine (without brain lysate), serving as a non-treated FRET-negative population. Each sample was run in triplicate. Representative images of the tau aggregates were captured using the ZOE Fluorescent Cell Imager (BioRad) in the GFP excitation and emission filters.

### Statistical analysis

Correlation analyses between PHF-tau burden quantification by immunohistochemistry, measurements of soluble tau contents as reported by Western blot, and IFD as reported by tau seeding assays were conducted using a Spearman correlation test. For non-parametric analyses, Kruskal-Wallis test was used to compare IFD in control, CTE and AD samples. Significance was set at *p* < 0.05. All statistical analysis and graphs were generated using GraphPad Prism v6.0 software (GraphPad Software Inc., La Jolla, CA).

## Results

### [^18^F]-AV-1451 phosphor screen autoradiography

**Fig. 1:**
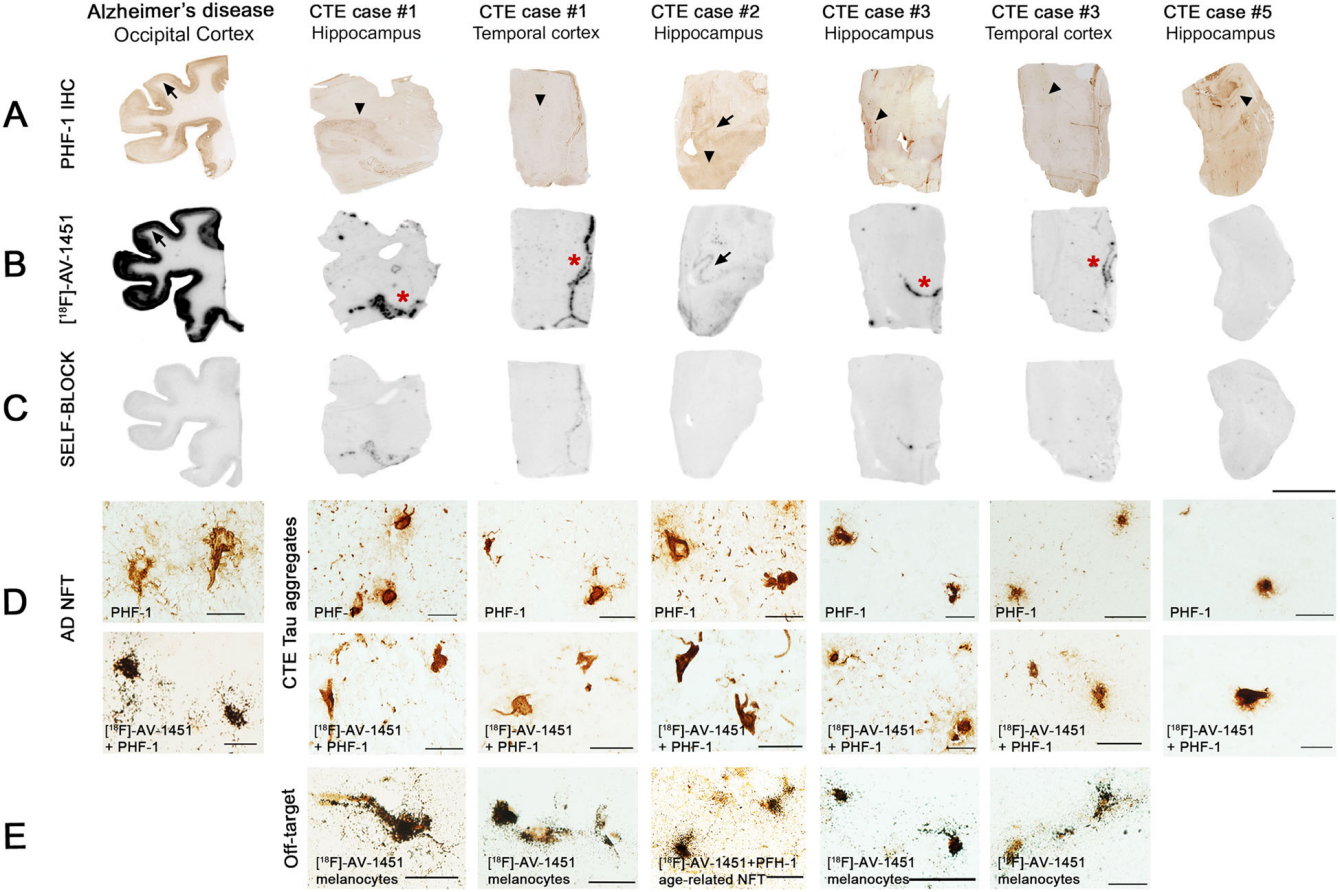
Representative images of immunohistochemistry with PHF-1 antibody (panel **a**), [^18^F]-AV-1451 phosphor screen autoradiography (panel **b**), self-block (panel **c**), and [^18^F]-AV-1451 nuclear emulsion autoradiography followed by PHF-1 immunostaining in AD (positive control) and CTE brains (panels **d** and **e**). A strong [^18^F]-AV-1451 binding signal was observed in cortical regions containing tangles in AD (panels **a** and **b** arrows). No [^18^F]-AV-1451 binding was detected in CTE slices containing abundant tau aggregates (panel **a** arrowheads) with the exception of strong binding to the choroid plexus in case#1 and to leptomeninges in cases #1 and #3 (panel **b** red stars) corresponding to *off-target* to leptomeningeal melanocytes. A weaker signal was present in hippocampus of case #2 (panel **b** arrow) where scarce classic AD NFTs (panel **a** arrow) were present along with abundant CTE tau aggregates (panel **a** arrowhead). The signal was blocked by adding unlabeled AV-1451 (panel **c**). [^18^F]-AV-1451 nuclear emulsion autoradiography confirmed a strong accumulation of silver grains colocalizing with classic NFTs in AD (panel **d**), and with incidental classic NFTs in the hippocampus of CTE case #2 (panel **e**). No detectable accumulations of silver grains were observed in association with CTE tau aggregates (panel **d**). Strong accumulation of silver grains also colocalized with leptomeningeal melanocytes in CTE cases #1 and #3 (*off-target binding*) (panel **e**). Scale bars = 1 cm (panels **a**, **b**, **c**) and 20 μm (panels **d** and **e**)

As expected, strong tracer binding was detected in tangle-containing regions from AD tissue used as positive control. However, no autoradiographic signal could be detected across multiple cortical regions known to contain tau aggregates in the 5 CTE cases (Fig. 1). The exception was the presence of strong tracer binding observed in a few isolated areas in 2 cases (case #1 HPC and TC, and case #3 HCP and TC) (Fig. 1). Adjacent sections stained with PHF-1 antibody and Thio-S showed that the autoradiography signal corresponded to *off-target* binding to leptomeningeal melanocytes and not to tau aggregates. Weaker binding was also noticed in the hippocampus from case #2 (the oldest individual in the CTE group, age 65) where some classic AD NFTs were present along with abundant CTE tau aggregates (Fig. 1).

**Fig. 2:**
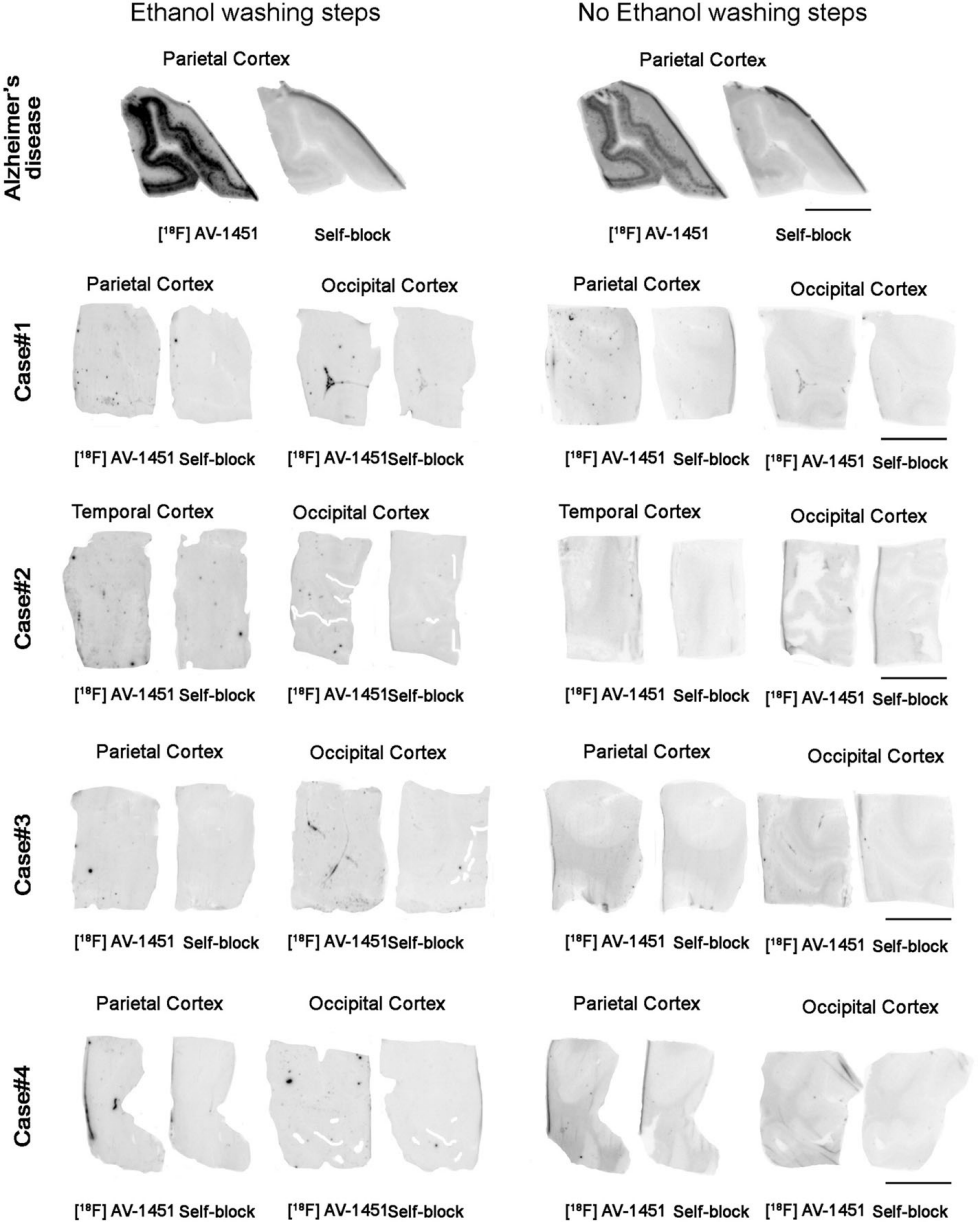
Representative images of phosphor screen autoradiography experiments with and without ethanol washing steps. Identical results were observed in the head to head comparison of both protocols using adjacent brain tissue slices. A strong [^18^F]-AV-1451 binding signal was observed in cortical regions containing tangles in AD but no [^18^F]-AV-1451 binding was detected in CTE slices with the exception of the *off-target* to leptomeningeal melanocytes. Scale bar = 1 cm

Parallel autoradiographic experiments performed on adjacent tissue slices and eliminating ethanol from the washing conditions yielded identical results (Fig. 2), ruling out the possibility that the ethanol washing steps may have removed some weaker tracer binding in these cases. A slightly higher non-specific background signal in the white matter was observed across cases when avoiding the use of ethanol in the washing steps.

### [^18^F]-AV-1451 high resolution nuclear emulsion autoradiography

To confirm the above observations and obtain cellular resolution, we performed autoradiography with photographic nuclear emulsion on histologic sections, followed by immunohistochemistry with appropriate tau antibodies. As expected, tangle-containing sections from cases of AD exhibited a strong concentration of silver grains colocalizing with NFTs. In tissue from cases of CTE, however, there was negligible accumulation of silver grains despite the presence of robust amounts of tau aggregates in the pathognomonic pattern of the disease (Fig. 1). Of note, abundant silver grains labeled the concomitant classic NFTs present in low amounts in the hippocampus of case #2, serving as an internal positive control for our autoradiographic experiments, but not the abundant CTE tau aggregates present in the same tissue material. In agreement with the above results from phosphor screen autoradiography experiments and our previously published observations [20, 21], silver grains strongly labeled leptomeningeal melanocytes (*off* target) found in two of the five CTE cases (case#1 and case #3) (Fig. 1).

### Total tau content by Western blot

**Fig. 3:**
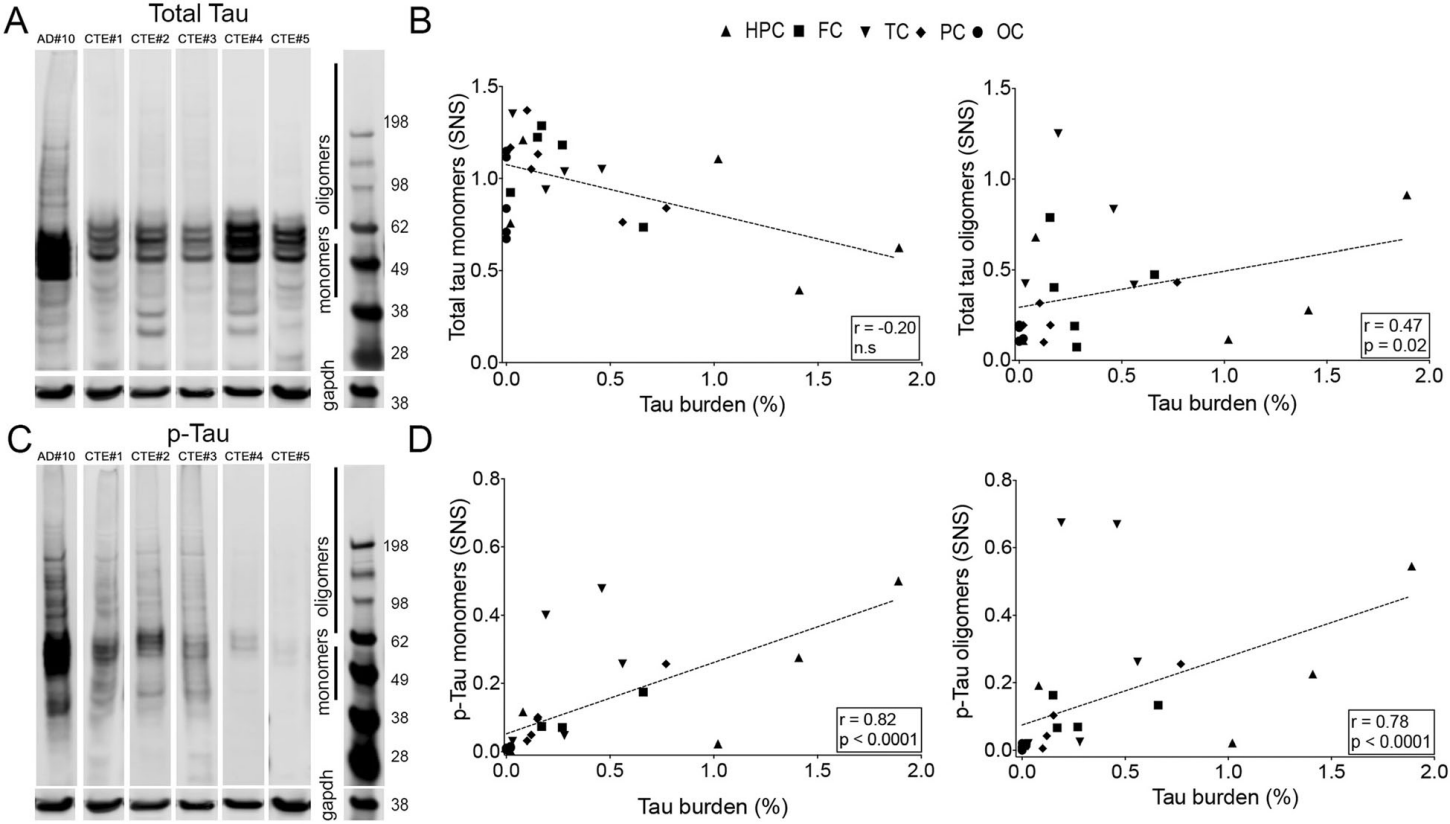
Representative images of Western Blot membranes probed with total tau (**a**) and PHF-1 (**c**) antibodies showing lower amounts of p-tau monomers and oligomers in CTE compared to AD brain samples. Significant correlations were found between total tau burden quantified on immunostained sections and levels of tau oligomers (**b**) and p-tau monomers and oligomers in CTE cases (**d**). Spearman *r* and *p* values are displayed on the graphs. Abbreviations: HPC: hipoccampus; FC: frontal cortex; TC: temporal cortex; PC: parietal cortex; OC: occipital cortex

Analysis of SNS fractions by Western blot revealed the presence of tau in the form of low molecular weight (monomeric) and high molecular weight (oligomeric) assemblies in CTE brains. Representative images of total tau and hyperphophorylated tau in ROIs from CTE cases as reported by Western Blot are shown in Fig. 3a and c. Levels of hyperphosphorylated tau were significantly lower in CTE cases compared to AD cases (hippocampus CTE vs. AD: pTau monomers 0.62 ± 0.70 vs. 2.56 ± 1.95, *p* = 0.04; pTau oligomers 0.46 ± 0.50 vs. 2.74 ± 3.41, *p* = 0.02; temporal cortex CTE vs. AD: pTau monomers 0.82 ± 0.69 vs. 5.32 ± 3.74, *p* = 0.03; pTau oligomers 0.77 ± 0.76 vs. 3.38 ± 2.82, *p* = 0.04; occipital cortex CTE vs. AD: pTau monomers 0.02 ± 0.006 vs. 5.46 ± 5.13, *p* = 0.03; pTau oligomers 0.01 ± 0.01 vs. 7.03 ± 7.22, *p* = 0.01) (Fig. 3c). A significant correlation was detected between burden of tau aggregates, measured in PHF-1 immunostained ROIs, and synaptic content of total tau oligomers (*r* = 0.47, *p* = 0.02, Fig. 3b) and phospho-tau monomers and oligomers in CTE cases (*r* = 0.82, *p* < 0.0001 and *r* = 0.78, *p* < 0.0001, respectively, Fig. 3d).

### Tau seeding assays

**Fig. 4:**
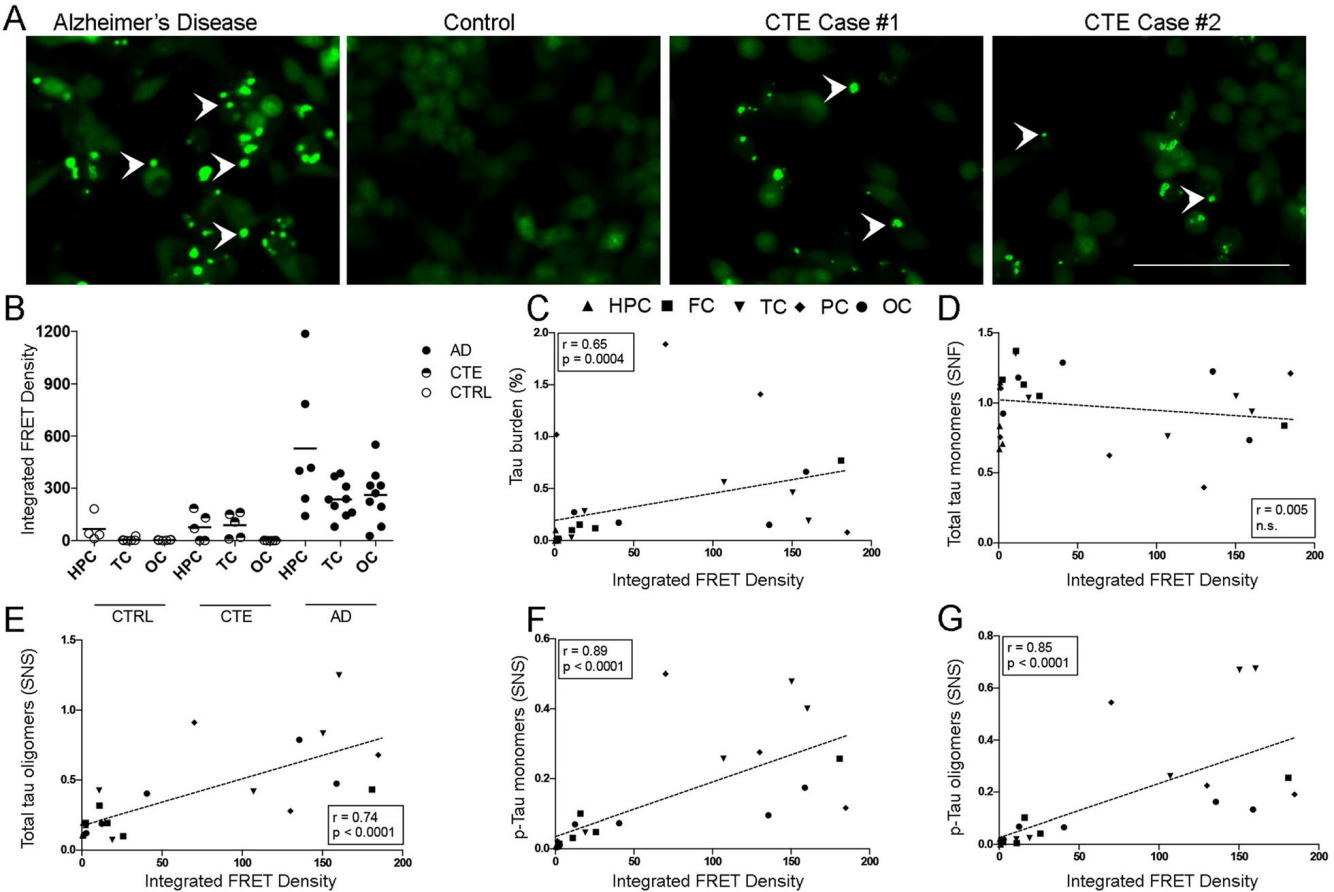
Representative confocal microscopy images showing tau aggregates in cells exposed to AD and CTE temporal cortex brain lysates (white arrow heads). No aggregates where detected in cells exposed to temporal cortex brain lysates from controls free of tau pathology (**a**). Quantification of IFD in ROIs from controls (*n* = 6), CTE (*n* = 5) and AD (*n* = 9) brain samples. IFD was significantly higher in AD than in CTE and control samples (*p* = 0.0009) (**b**). IFD values showed a significant correlation with total tau burden quantified by IHC (**c**) and with total levels tau oligomers (**e**), p-tau monomers (**f**) and p-tau monomers (**g**) but not with total tau monomers (**d**). Spearman *r* and *p* values are displayed on the graphs. Abbreviations: HPC: hipoccampus; FC: frontal cortex; TC: temporal cortex; PC: parietal cortex; OC: occipital cortex; IFD: integrated FRET density; IHC: immunohistochemistry. Scale bar = 100 μm

Tau seeding activity across the ROIs in CTE brains was significantly higher than in control brains free of tau-containing lesions but significantly lower than in AD brains (*p* = 0.0009) (Fig. 4a-b). Representative images of tau seeding activity assays are depicted in Fig. 4a. Of note, a substantial patient to patient variability was noted in both groups, CTE and AD (Fig. 4b). Tau seeding activity in CTE cases closely correlated with the total burden of tau aggregates (%) measured in PHF-1 immunoreactivity (*r* = 0.65, *p* = 0.0004, Fig. 4c), as well as with the levels total tau oligomers (*r* = 0.74, *p* < 0.0001, Fig. 4e) but not total tau monomers (*r* = 0.005, n.s, Fig. 4d), and with levels of soluble phospho-tau monomers and oligomers (*r* = 0.89, *p* < 0.0001 and *r* = 0.85, *p* = 0.0001, respectively, Figs. 4f-g), as measured by Western blot.

## Discussion

We have examined the postmortem regional- and substrate-specific binding patterns of tau PET tracer [^18^F]-AV-1541 in multiple ROIs from five autopsy-confirmed CTE cases. Our observations are derived from [^18^F]-AV-1451 sensitive autoradiography, quantification of tau burden on immunostained sections, biochemical analysis of soluble tau content by Western blot and in vitro tau seeding activity assays. In agreement with our previous observations, high resolution autoradiography experiments revealed strong binding of [^18^F]-AV-1451 to NFTs in AD [20, 21], but negligible binding to tau aggregates in CTE brains. A strong [^18^F]-AV-1451 binding to incidental leptomeningeal melanocytes present in two of the five CTE cases further confirmed tracer *off-target* binding to this biological substrate [20, 21]. These observations, along with the lack of correlation between [^18^F]-AV-1541 autoradiographic regional binding at postmortem and the topographical distribution of tau aggregates or multiple other tau measures in these cases of CTE, suggest that [^18^F]-AV-1451 may not have sufficient sensitivity to reliable detect and quantify tau pathology in CTE by in vivo neuroimaging particularly when confounding AD lesions are present in the context of aging.

As in other neurodegenerative conditions, the development of novel fluid and neuroimaging biomarkers potentially capable of facilitating an accurate diagnosis of CTE and of monitoring disease progression has become a priority in this research field [37, 42]. While tau PET tracer [^18^F]-AV-1451 has recently shown great promise as a surrogate biomarker for tau-containing lesions in AD through its binding to NFTs with PHF conformation, the potential utility of this ligand in assessing the burden of the distinct tau-containing lesions of CTE remains uncertain. To date, only very limited data from human in vivo [^18^F]-AV-1451 PET imaging studies in patients with clinically suspected CTE have been published and they all, unfortunately, lack neuropathological confirmation of the diagnosis. Mitsis et al. reported a 71-year old retired NFL football player who had experienced multiple concussions and presented with progressive cognitive impairment in his 60s. [^18^F]-Florbetapir PET was negative, excluding significant concomitant amyloid deposition, while [^18^F]-AV-1451 PET showed increased uptake localized in globus pallidus, putamen, hippocampus and the substantia nigra [29]. However, we and others have described a nearly identical pattern of in vivo retention in elderly controls [17], that seems heavily influenced by the non-specific retention of this tracer in basal ganglia and its *off-target* to neuromelanin-containing neurons in the substantia nigra [21]. Dickstein et al. reported another 39-year old retired NFL football player with a history of multiple concussions who experienced cognitive decline, irritability and emotional lability in his 30s. [^18^F]-Florbetapir PET was negative and [^18^F]-AV-1451 showed increased retention in midbrain, globus pallidus and hippocampus, and also at gray-white matter junctions in multiple cortical areas, mirroring the described postmortem distribution of CTE tau lesions [7]. It should be noted though that, in the absence of a validated threshold for defining signal with [^18^F]-AV-1451, the authors relied on the value commonly used for the amyloid PET tracer florbetapir and thus, these results must be interpreted with caution [18]. Very recently, another study by Stern et al. has reported a significantly higher [^18^F]-AV-1451 in vivo retention at the group level in three brains regions (bilateral superior frontal, bilateral medial temporal, and left parietal) in a cohort of 26 former National Football League (NFL) players with cognitive and neuropsychiatric symptoms compared to 31 asymptomatic men with no history of traumatic brain injury [36]. It remains unclear whether such differences were also present at individual level. Of note, the average in vivo retention values observed in the group of NFL players in that study were quite modest (SUVR values 1.09–1.12), with the exception of the medial temporal lobe (1.23); most fell within the range of in vivo retention values reported in normal elderly controls in multiple other studies [31]. Intriguingly, no association between [^18^F]-AV-1451 in vivo retention and scores on cognitive and neuropsychiatric tests could be demonstrated. The potential presence of age-related tau-containing lesions in the medial temporal lobe and tracer *off-target* binding to old hemorrhages, which are quite common after brain trauma, are potential confounders when interpreting these results.

All of the 5 CTE cases studied here demonstrated immunohistochemically confirmed abundant tau aggregates in a distribution consistent with the consensus diagnostic criteria. Importantly, we carefully selected the CTE cases included in this study ruling out the presence of substantial concomitant AD pathology as a confounder in our autoradiography experiments given the known strong affinity of [^18^F]-AV-1451 to classic AD tau tangles. Our phosphor-screen autoradiography experiments, showed negligible binding of [^18^F]-AV-1451 in brain regions with a high burden of tau lesions apart from *off-target* binding to leptomeningeal melanocytes present in two of the five cases. In contrast, tangle-containing sections from AD brains, included here as positive control, exhibited strong [^18^F]-AV-1451 binding signal as previously reported by us and others [19–22, 33, 41]. In agreement with these findings, high resolution nuclear emulsion autoradiography experiments showed high concentrations of silver grains colocalizing with NFTs in AD brains and incidental extracutaneous leptomeningeal melanocytes but no detectable silver grain accumulation colocalizing with CTE tau aggregates.

The possibility that the use of ethanol in the washing steps included in our traditional autoradiography protocol may have removed some weaker tracer binding was carefully ruled out by performing experiments which avoided the use of ethanol or other solvents in the washing conditions. Those parallel autoradiography studies yielded identical results.

The pathological tau burden in the CTE cases, quantified on immunostained sections containing multiple ROIs, significantly correlated with the levels of soluble p-tau monomers and oligomers, as assessed by Western blotting, and with tau seeding activity in the same ROIs. These measures of pathologic tau aggregates, however, were substantially lower than those found in a series of 9 AD cases that were used here for comparison. Intriguingly, a previous study by Woerman et al. reported increased tau seeding activity in CTE when compared to AD brains [40]. We believe that the discrepancies between that and the present study may be related to differences in sampling, given the highly focal nature of CTE [40], and/or the potential presence of concomitant AD pathology, a common finding in this condition [26], in older individuals with CTE. As noted above, our studies specifically excluded coincident neuropathologic processes, including the presence of substantial AD pathology, as a potential confounder through careful case selection.

Our results suggest that [^18^F]-AV-1451 differs in its affinity for the tau aggregate-containing lesions of CTE and AD, despite both being comprised of 3 and 4 repeat isoforms of tau. Importantly, recent studies based on cryo-EM have elegantly demonstrated the existence of distinct conformers of assembled tau in different tauopathies [9, 10]. That work has demonstrated that, despite the presence of both 3 and 4 repeat isoforms, the tau filament structures in CTE are distinct from those of AD, as well as from the 3 repeat isoform containing tau aggregates of Pick disease. Even though similarly to AD, all six tau isoforms assemble into filaments in CTE, a conformation of the β-helix region creates a hydrophobic cavity that is absent in tau filaments in AD. Moreover, filaments in CTE have distinct protofilament interfaces to those of AD [10] The distinct conformations of tau filaments in different tauopathies may not only explain the phenotypic and neuropathologic diversity of these disorders but also underlie the differential affinity of PET ligands, like [^18^F]-AV-1451, for tau aggregates in AD and non-AD tauopathies. This may, in part, reflect the process by which this compound was identified as potential tau imaging agents screening using cortical homogenates from AD brain tissue rich in NFT as the binding target. A potential limitation of the current study is the lack of females in the CTE group.

## Conclusions

In conclusion, the results from this study further favor the idea that tau tracer [^18^F]-AV-1451 binds with high affinity to tau aggregates in AD brains and that now established *off-target* binding must be carefully taken into account when interpreting its behavior in vivo. Our data also indicate that [^18^F]-AV-1451 exhibits relatively low binding affinity to tau inclusions in CTE and suggest that this ligand may have a limited utility for the reliable detection and quantification of tau lesions in this non-AD tauopathy. The combination of the lower pathological tau load in CTE brains when compared to AD and the apparent differential affinity of this imaging agent for disease-specific molecular conformations of tau filaments suggest that [^18^F]-AV-1451 may not be an optimal agent to use for assessment of CTE, particularly in older individuals where the presence of concomitant AD pathology is a frequent finding. Further neuroimaging-pathologic correlation studies are needed to accurately interpret what in vivo [^18^F] AV-1451 PET positivity means. Although we cannot rule out with absolute certainty that [^18^F] AV-1451 may exhibit some weak binding affinity for tau aggregates in CTE, we believe that selective screening using different tau conformations as binding targets may result in better and more reliable PET tracers for CTE and other non-AD tauopathies.

## Data Availability

Original slides and diagnostic material are retained. There are no novel reagents or materials for others to request.
